# Seroprevalence, frequency of leptospiuria, and associated risk factors in horses in Kansas, Missouri, and Nebraska from 2016-2017

**DOI:** 10.1371/journal.pone.0206639

**Published:** 2018-10-29

**Authors:** Amanda C. Trimble, Christopher A. Blevins, Laurie A. Beard, Ashley R. Deforno, Elizabeth G. Davis

**Affiliations:** Department of Clinical Sciences, College of Veterinary Medicine, Kansas State University, Manhattan, Kansas, United States of America; Universidade Federal de Pelotas, BRAZIL

## Abstract

Leptospirosis is a worldwide veterinary and public health concern, and well recognized infectious disease of horses. Seroprevalence rates vary with geography, but many studies have confirmed a high exposure rate. The correlation between seropositivity and shedding status has not been made in horses, however. The aims of this study were to use semi-nested PCR on urine from apparently healthy horses to determine period prevalence of leptospiral shedding and to correlate these findings with MAT results to establish associations with client based survey data regarding horse management and environment. Serum and free-catch urine were collected from 204 healthy horses between May 2016-December 2017. Serum was used to determine GGT, creatinine concentrations, and six serovar MAT. Urine samples were submitted for PCR testing of leptospiral 23S rRNA. Client consent and survey data were collected for all subjects. Potential risk factors included drinking water source, exposure to livestock and dogs, geographical location, season, and precipitation. Two horses were positive on urine PCR for leptospirosis (shedding prevalence 1%), yet only one had a high reciprocal MAT titer of ≥ 800. Both horses were negative on urine PCR one month later without treatment. Approximately 77% of horses (157/204) were seroreactive (MAT reciprocal titer ≥ 100) with titers to serogroup Australis detected more frequently than others (47.5%; (97/204)). Apparently healthy horses infrequently shed *Leptospira* spp. in urine, yet seroreactivity in clinically normal horses is high (77%), confirming high exposure rates to *Leptospira* spp. in the Central Midwest.

## Introduction

Leptospirosis, one of the most important worldwide zoonotic diseases, can present in horses in a number of ways. Although exposure rate is high based on seroprevalence studies, most horses are subclinically infected. Horses may manifest nonspecific clinical signs, such as anorexia, lethargy, fever, and icterus [1–2] that do not warrant diagnostics for leptospirosis. Disease syndromes frequently associated with leptospirosis in horses that carry a higher index of suspicion for the disease include equine recurrent uveitis (ERU), acute renal failure, sporadic abortions, placentitis, stillbirths, and, more recently, pulmonary hemorrhage and hemolysis [2–9]. As many clinical signs associated with leptospirosis are non-specific, disease in horses may occur more frequently than is diagnosed, and exposure to *Leptospira* spp. may be more prevalent than was previously thought. The incidence and importance of equine leptospirosis has not been extensively studied to date.

Epidemiological studies typically employ the microscopic agglutination test (MAT) to determine a seroprevalence rate. In these serological survey studies, the MAT provides information on exposure rates and suspected infecting serogroups in the geographic region being studied, but tells nothing about the carrier or shedding status of the horses. In a recent study by Zoetis LLC., the reported seroprevalence of leptospirosis in horses was 76.2% in the Midwestern United States, prompting the development of a commercially available equine vaccine. This study further showed that 75% of healthy horses have been exposed to at least one leptospiral serogroup [10]. Observation or detection of leptospires in urine by dark-field microscopy, culture, or polymerase chain reaction (PCR) provide direct evidence of the carrier or shedding status of horses [11].

The use of PCR to detect the presence of pathogenic leptospires in urine, fetal membranes, and aqueous/vitreous humor has been reported in horses to definitively diagnose leptospirosis as the cause of disease and identify leptospiral shedding [4, 12–15]. A study from Brazil demonstrated a seroprevalence rate (reciprocal titers ≥200) in horses of 39.8% (55/138) and identified the presence of leptospires by PCR in 50 of 138 (36%) urine samples [14]. Interestingly, 52% (26/50) of the horses that had a positive PCR on urine were seronegative, suggesting that serologic testing is a poor predictor of urinary shedding.

To our knowledge, an investigation of urine shedding of *Leptospira* spp. by asymptomatic horses in the Central Midwest using PCR has not been performed. This information would be of practical use for determining carrier prevalence in a specific geographical area, as well as increasing awareness of the potential for infectious and zoonotic spread by horses in the environment and to their owners.

### Objective

The objectives of this study were to evaluate the seroprevalence, frequency of leptospiral shedding in urine, and environmental risk factors for seropositivity of asymptomatic, apparently healthy horses in Kansas, Missouri, and Nebraska.

### Hypothesis

Our hypotheses were that seroprevalence would be high in the study population, urinary shedding of pathogenic leptospires would be lower than seroprevalence and not predicted by serological titers, and that horses stabled outside, living near fresh water sources such as ponds, and living in close proximity to dogs and/or livestock would be at greater risk for seropositivity and urinary shedding of pathogenic leptospires.

## Materials and methods

### Ethical approval

The study complied with all Institutional Animal Care and Use Committee of Kansas State University regulations and was approved by the committee prior to data collection (IACUC #3727).

### Subject selection

The study was designed as a cross-sectional prevalence study representing horses of mixed breeds and ages, owned by Kansas State University, the Animal Science Unit Equine herds, and clients of the Kansas State University Veterinary Health Center (VHC) (KS, NE, MO). This study was performed over 19 consecutive months to account for temporal bias. Apparently healthy horses presented for pre-purchase exams, dentals, and annual vaccines, as well as volunteered animals. Horses were evaluated with a physical examination performed by a licensed veterinarian, and were deemed healthy.

Power analysis was performed prior to sample collection to determine appropriate sample size (126–153 total needed), based on expected prevalence estimates (3–5% prevalence) for urine shedding, with all other assumptions held constant, a desired confidence of 0.95, and a precision of 0.05 [16–17].

### Inclusion criteria and sample collection

Horses were excluded if there was any history of renal disease, ERU, recent abortions (in the last six months), liver failure, pulmonary hemorrhage, or they had ever received a leptospirosis (on or off label) vaccine. Samples (one serum sample via aseptic venipuncture and one free catch urine sample) were collected on the same day either at the VHC or at the horse’s home environment. All samples were either submitted to the Kansas State University Diagnostic Laboratory (Manhattan, KS, USA) directly, or submitted within 48 hours post collection after being stored at 4°C. Blood was collected into tubes without anticoagulant. Blood was either sent to the laboratory uncentrifuged or left to clot for 30 min before centrifugation at 2000 × g for 10 min and subsequent serum separation. Serum samples were submitted for measurement of creatinine and gamma glutamyl transferase (GGT), and performance of the MAT, while the urine sample was submitted for identification of pathogenic leptospires by PCR. Urine samples were collected using a free catch method. If the horse did not void urine with the use of alpha-2 agonists or during the exam, a single dose of furosemide 5% was given intravenously at a dose of 0.5mg/kg bwt.

Enrollment criteria included permission and written consent. Using an owner reported survey, we recorded environment and management practices for each horse including: street address (to allow for geographical grouping), stable vs. pasture environment, proximity to water sources, where drinking water is obtained (city, rural, well etc.), recent rainfall on the property, and proximity and contact with any livestock or dogs. All positive results were reported to the owners and recommendations on management and handling of these horses were given on an as needed basis. Maps were generated using https://maps.google.com. Rainfall data was determined using https://www.wunderground.com/history.

### Creatinine and GGT

Serum creatinine and GGT were determined using the Cobas 6000 analyzer series, Roche diagnostics USA.

### Microscopic agglutination titers

Serum samples were collected from each horse and submitted to the Kansas State Veterinary Diagnostic Laboratory-Serology Laboratory for MAT determination of titers to serogroups Canicola, Pomona, Icterohaemorrhagiae, Australis, Sejroe, and Grippotyphosa based on the National Veterinary Services Laboratory's (NVSL) protocol (Ames, Iowa, USA). In brief, spectrophotometry is used to detect leptospirosis cell counts to the six serovars (representative of serogroups): Canicola, Pomona, Grippotyphosa, Icterohaemorrhagiae, Hardjo, and Bratislava. The strains selected are grown in liquid leptospiral culture medium and used as leptospiral antigens to do a transmittance percentage. The number of antigens used is determined and a screening test may be performed with a 1/50 serum dilution. Quality control is performed. A volume of each antigen, equal to the diluted serum volume, is added to each well, making the final serum dilution 1/100 in the screening test and these microtitration plates are incubated for 1.5 hours. End-point titers are determined. The plates are examined with dark-field microscopy. Results were reported as seronegative if the reciprocal titer was <100.

### Polymerase chain reaction

Free catch urine samples were collected and stored in a sterile plastic container. Urine was refrigerated for a maximum of 48 hours prior to submission to the Kansas State University Diagnostic Laboratory. The PCR methodology used is briefly described here (adapted from Harkin et al. 2016 [18]):

### DNA Isolation

From each urine sample that was submitted, 1.8 ml of urine was centrifuged at 15,000 *g* for 20 minutes, the supernatant was discarded, and the pellet re-suspended in 140 μL of sterile phosphate buffered saline (PBS). DNA isolation was performed on the suspended pellet by use of the QIAamp viral RNA kit (Qiagen Inc,) following manufacturer’s instructions (spin protocol).

### Polymerase chain reaction assay

For the initial PCR 2.5 μl of each DNA template was added to 22.5 μl of master mix (1 X PCR Buffer B, 4.0 mM MgCl_2_, 800 nM dNTP mix, 2.5 U of Taq polymerase (Fisher Bioreagents); 0.5% bovine serum albumin (Sigma-Aldrich); and 400 nM each of L737 forward primer (5’–GAC CCG AAG CCT GTC GAG– 3’) and L1218 reverse primer (5’ GCC ATG CTT AGT CCC GAT TAC– 3’)) [19–20]. The reactions were performed on a standard thermal cycler for 30 cycles at 95°C for 30 seconds, 60°C for 1 minute and 72°C for 1 minute. For each run, a no-template control of TE buffer (10mM Tris pH 8.0, 1mM EDTA), DNA extracted from a saprophytic control, *L*. *biflexa* serovar Patoc, and a pathogenic control, *L*. *interrogans* serovar Canicola, each diluted 1:5,000, were used as DNA controls for the assay.

After centrifugation of the initial PCR reactions, 1.0 μl of each reaction was used as template for a second semi-nested PCR in 25 μl reaction volume. The master mix for this step was identical as for the initial PCR except for the following: L1218 reverse primer was replaced with Lep2R reverse primer (5’–TTA TCC CCC GTA GTC TGA CTG C– 3’) and a dual labeled Taqman probe (LEP883F-FAM Probe (5’– 56-FAM/CTC CGA AAT AGG TTT AGG CCT AGC GTC AG/BHQ-1–3’)) was added. The PCR was performed on the SmartCycler II, (Cepheid) real-time PCR system using the following protocol: 94°C for 1 minute with the optical sensor off, then 45 cycles at 94°C for 10 seconds, 60°C for 20 seconds, and 72°C for 30 seconds, with the optical sensor on during the 72°C extension step. The initial and final fluorescence were recorded with the background off. With the background off, the initial and final raw fluorescence value for each sample was recorded, with the difference in these values calculated as the change in fluorescence. A positive sample was defined as a positive change in fluorescence greater than 50 fluorescent units. The PCR assay as reported here has a sensitivity of 93% and specificity of 100% (unpublished data).

### Statistical analysis

All data was collected into Microsoft Excel. The prevalence of positive urine PCR and MAT seropositivity were calculated. In order to examine the different reported titer levels indicating active infection on MAT seropositivity, seroprevalence was defined at both reciprocal titers of ≥100 and ≥800. Risk factor analysis was restricted to MAT seropositivity as outcome measure because only two horses tested positive on PCR. Risk factor analysis was performed using 95% confidence intervals (95% CI) by the modified Wald method. Logistic regression models (R, Commercial Statistical Software) were performed. The Chi-square test and odds ratios were used to assess associations between seroreactivity and potential risk factors using reciprocal MAT values of ≥100 and ≥800 with a multivariate model. Values of p ≤ 0.05 were considered statistically significant.

## Results

Samples were collected from total of 204 horses of mixed age, breed, and sex between May 2016 and December 2017. Sixty-nine mares, 103 geldings, and 32 stallions were represented. Age ranged from 1–40 years, with the majority of horses being between the ages of 1–20 years (median-13 years). Quarter horse type breeds were most represented in this population (124/204; 61%), followed by Thoroughbreds (16/204;7.8%), Warmbloods (15/204;7.4%), and 15 other breeds and crossbreeds. Horses were located in 54 different zip codes spread between Kansas, Nebraska, and western Missouri (Kansas State University-VHC service area).

Based on the client survey, the main housing, primary water source, known contact with other domestic animals, presence of rain in the previous week, and season in which the samples were collected are shown in Table 1. Although treated water was the primary water source for 70% of the horses, 31.8% of all horses had creeks, ponds, or streams on the farm that they lived. Despite efforts to collect equal numbers of samples from each season to account for temporal bias, the majority of samples were collected in the fall.

**Table 1:** Client based survey categorical responses concerning environmental conditions and management practices of the 204 horses most likely to affect leptospirosis carrier and exposure status based on its biological nature.

	Number of Horses (n/204)	Percentage (%)
**HOUSING**		
Stable (Full)	21	10.29
Pasture (Full)	74	36.27
Mixed (Stable + Pasture)	67	32.84
Dry Lot	42	20.59
**PRIMARY WATER SOURCE**		
Treated (City + Rural)	143	70.00
Well	56	27.45
Pond	3	1.47
Other	2	0.98
**CONTACT W CATTLE, SHEEP, DOGS**		
Yes	151	74.02
No	53	25.98
**RAIN PAST 7 DAYS**		
Yes	177	86.76
No	27	13.24
**SEASON**		
Spring	44	21.57
Summer	39	19.12
Fall	85	41.67
Winter	36	17.65

Two of 204 horses were positive on urine PCR for leptospirosis, which is an overall shedding prevalence of 1% for this study population. The PCR-positive horses were sampled on June 30^th^, 2016 (Horse 1), and on October 28^th^, 2016 (Horse 2). Both horses were from the same zip code in Kansas, but were from different farms. Neither horse had traveled out of state in the previous 6 months, and were both maintained on pasture full time, drank from treated water sources, and had access to cattle and/or dogs. The horse sampled on June 30 lived with 3 other horses (all included in study), had a creatinine of 0.8 mg/dL, a GGT of 7 U/L, and there had been 2.43 inches of rainfall in the area in the preceding week. The PCR cycle threshold (Ct) for this horse was 15.45. Horse 2 lived on a property with 25 other horses (not all included in study), had a creatinine of 1.1 mg/dL, and GGT of 18 U/L, and there had been 0.79 inches of rainfall in the area in the last 7 days. The PCR Ct for this horse was 12.55. Horse 1 had a reciprocal titer of 200 to serogroup Icterohaemorrhagiae and 100 to serogroups Pomona and Sejroe on initial sampling, but was seroreactive to only serogroup Australis with a reciprocal titer of 200 one month later without therapy being administered. Horse 2 had a reciprocal titer of 25,600 to serogroup Icterohaemorrhagiae and a reciprocal titer of 3,200 to serogroup Australis at initial sampling. No therapy was administered and one month later, the reciprocal titers were 6400 to serogroup Icterohaemorrhagiae and 3,200 to serogroup Australis (lesser reciprocal titers were seen to serogroups Canicola (800 and 400, initially and at one month respectively) and serogroups Pomona and Grippotyphosa (100 only at initial testing)). Although the MAT and positive PCR for Horse 2 were indicative of an acute infection, the horse displayed no outward clinical signs of leptospirosis, nor renal, hepatic, pulmonary, or other organ compromise. No horses had evidence of renal or hepatic compromise based on clinicopathologic values or by the veterinarians performing the clinical examinations.

Overall seroprevalence was 77.0% (157/204) at a reciprocal titer of ≥100 and 14.7% (30/204) at a reciprocal titer of ≥800. The highest reciprocal titer was 25,600 in one horse to serogroup Icterohaemorrhagiae, followed by serogroup Pomona at 6400 in 2 horses. When considering only the highest reciprocal titer for each horse as the most likely infecting serogroup, the most prevalent infecting serogroup was Australis (30.5% (48/157)), followed by serogroups Grippotyphosa (25.4% (40/157)) and Icterohaemorrhagiae (17.8% (28/157)). A highest titer to serogroup Pomona was uncommon (3.2% (5/157)), but these 5 horses had significantly elevated reciprocal titers of 3,200 (n = 3) and 6400 (n = 2). Equivalently high reciprocal titers to serogroups Australis with Icterohaemorrhagiae (n = 10) and Australis with Grippotyphosa (n = 6) accounted for an additional 10.2%. The remaining 12.9% was scattered among serogroup Canicola (1.3%) and a mix of 10 other combinations. The highest reciprocal titer to serogroup Australis was 3,200 (n = 3) and 5 horses had a reciprocal titer of 1,600.

To further analyze potential risk factors, modified Wald tests were performed to determine whether the risk factors highlighted in Table 1 were associated with a horse having a reciprocal titer of ≥100 or ≥800. No significant correlations were detected when the cut-off was classified as test-positive reciprocal titer ≥800. However, season (*p = 0*.*004*) and rainfall in the past week (*p = 0*.*035*) were significantly associated with the odds of having a reciprocal titer ≥100 cut-off. Multiple logistic regression using the same variables showed that the summer and fall seasons, as well as rainfall in the last seven days, were significantly associated with the odds of horses having a reciprocal titer ≥100 (Table 2). Horses who experienced rainfall within the past 7 days had 3.4 times (95% CI: 0.123; 0.927) greater odds of having a reciprocal titer ≥100 than those who did not experience rain. Horses that were sampled in the summer or fall had 8.46 and 4.10 times greater odds, respectively, to have a reciprocal titer ≥100 than those collected in the spring (Table 3). Only 20.9% of horses with access to ponds, stream, and creeks were seronegative.

**Table 2:** Logistic regression model of horses with one or more reciprocal MAT titers ≥100 and potential risk factors for leptospirosis exposure.

HOUSING (ref stable)	Estimate	Std. error	Z value	P-value
Pasture	-0.7328	0.7442	-0.985	0.32480
Mixed	-0.8026	0.7402	-1.084	0.27821
Dry Lot	-1.1193	0.7953	-1.407	0.15932
**PRIMARY WATER SOURCE (ref city)**				
Rural	-0.4095	0.4957	-0.826	0.40881
Well	-0.7718	0.5407	-1.427	0.15349
Pond	-1.4722	1.3598	-1.083	0.27894
Other	13.0547	1017.3132	0.013	0.98976
**CONTACT W CATTLE, SHEEP, DOGS (ref yes)**				
No	0.2367	0.4918	0.481	0.63033
**RAIN PAST 7 DAYS (ref yes)**				
No	**-1.0870**	**0.5159**	**-2.107**	0.03512*
**SEASON (ref spring)**				
Summer	**2.1356**	**0.6803**	**3.139**	0.00169**
Fall	**1.4112**	**0.4706**	**2.999**	0.00272**
Winter	1.0238	0.5727	1.788	0.07384

Note

* represents p-value <0.05 and

** represents p-value <0.01.

**Table 3:** Odd Ratios (95% confidence interval) of season from multivariate model of seroreactivity defined as a reciprocal MAT titer of ≥ 100.

Reference	Spring	Summer	Fall	Winter
**Spring**		8.46 (2.23, 32.11)**	4.10 (1.63, 10.31)**	2.78 (0.91, 8.55)
**Summer**			0.48 (0.14, 1.71)	0.33 (0.09, 1.27)
**Fall**				0.68 (0.25, 1.87)

Note

* represents-value less than 0.05;

** represents p-value less than 0.01.

## Discussion

The objectives of the present study were to determine whether apparently healthy horses were shedding leptospiral bacteria in their urine and acting as potential carriers using urine PCR. Our secondary objectives were to determine the seroprevalence of leptospirosis in horses in the Central Midwest and to identify potential risk factors for equine exposure that could be used as a component of a prevention and control strategy for human and other animal exposure.

In the present study, there was a urinary shedding rate of 1% from asymptomatic horses. The overall prevalence from this population was expected to be low, based on previous studies from other species in non-endemic areas. In dogs, between 1.5–8% of urinary leptospiral shedding from asymptomatic canids has been reported [21–23]. Interestingly, in our study, one PCR positive horse had extremely high MAT titers, consistent with active infection [24]; however, was not displaying any outward signs of clinical infection, indicating that subclinical infection can potentially be a real issue with disease spread and zoonosis. Since both seronegative and seroreactive animals can shed the bacteria, direct detection of leptospires in urine (by culture or PCR) is an important tool for earlier leptospiral detection, a successful control program, and may better guide vaccination protocols.

In our study, 77% of horses were seropositive (reciprocal MAT titer ≥ 100) to at least one of the 6 serogroups tested, which is consistent with the previously reported seroprevalence for the Midwestern US (76.2%) [10]. The panel of serovars analyzed in this study included locally occurring serovars found in dogs, cattle, and horses. After infection, antibodies (IgG and IgM) can persist for years or even during the entire lifespan of an animal [25]. As Blatti and others concluded from a similar study [26], we also suspected that the high seroprevalence in these healthy horses suggests that horses are often exposed to or may be carriers of *Leptospira* spp. and never develop clinical signs. This is most likely due to exposure to host-adapted serovars of *Leptospira* spp., leading to an inapparent infection in their respective hosts. In contrast, some horses may be seronegative but PCR positive. This scenario may be due to being infected by a serovar not detected on that specific MAT, potential immunosuppression and an inadequate antibody response, testing an infected animal where the serovars are localized to an immunologically protected site, or testing prior to seroconversion [20]. Previous clinical leptospirosis infection or vaccination leading to the development of antibody production in our sample population is unlikely, as any of these horses were excluded prior to sampling and analysis.

In a study by Blatti and others from 2011 [26], risk factors for increased seroprevalence were increasing age (*p = 0*.*006*), being a mare (*p = 0*.*001*), being a pony (*p = 0*.*028*), increasing duration spent on pasture per day, as well as seasonal variation in seropositivity with the prevalence being higher in summer and autumn, and lower in winter and spring (*p = 0*.*003*). While we did appreciate increased seroprevalence in the summer and fall seasons, we also saw increased seroprevalence with reports of rain in the previous week, and we must interpret these results with caution. We did not experience significant flooding during the study period, even though most horse owners (177/204; 86.76%) reported rain. The exact amount of rain in each location for each seropositive horse was not documented. A future study looking at rainfall over a longer period of time on the individual sample site level may also be of interest. Summer and autumn having higher seroprevalence seems to be a frequently reported risk factor, as we saw in our study. Thus, we are confident reporting that horses are 8.4 times as likely in the summer and 4.1 times as likely in the fall to be seroreactive compared with horses in the spring. The increased seroprevalence in spring and fall is likely due to longer survival and infectivity of leptospires in the environment in warm and humid climate conditions. Pond water as the primary water source was low at 1.5%, but 31.8% of the horses had creeks, ponds, or streams on the farm that they lived. Only 20.9% of horses with reported ponds, stream, and creek on their property were seronegative, however it was unknown how many horses had direct contact with these water sources. We expected that water sources, increased time spent on pasture, and contact with potential leptospirosis reservoir species (dogs, cattle, sheep) would potentially have a significant effect on seroprevalence based on the biology of the bacteria, however these results were not significant in our study. In another study by Hamond and others [27], as well as one by Wangdi and others [28], it was also found that different types of water sources did not have any influence on the status of horses being seropositive to *Leptospira* spp., which could be an interesting topic to explore further. No significant findings were appreciated in our study population with regards to risk when the positive MAT titer was defined as the reciprocal of ≥ 800. This could potentially be due to smaller sample size (only 14.7% of horses), this population being considered healthy and not having active or clinical leptospirosis, or there truly being no increased risk of having a reciprocal titer of greater than or equal to 800 with the variables evaluated.

Serogroup Australis (represented by serovar Bratislava) was most commonly observed in this study: 47.5% with all reactive serovars of every horse included (reciprocal titers of ≥100), 30.5% as only the highest reciprocal titer (≥100) from seropositive horses, and 18 horses having a reciprocal titer of ≥800. In a study in dogs in Switzerland, serovars Australis and Bratislava, both belonging to serogroup Australis, were also associated with about half of the seroreactors. In Switzerland, infections with this serogroup have been known to cause acute clinical infections, and are suspected to be transmitted by hedgehog reservoir hosts [27]. While many previous reports from the US have implicated Pomona as the most common serovar, finding Australis in our study was not that surprising. In a study in South Africa, the most common serovar in all three provinces analyzed was Bratislava (Australis), and other studies performed around the world have reported the seropredominance of this serovar in their surveys in horses [17, 27]. Interestingly, in the current study, we noted that after Australis, depending on how we defined seroreactivity had a profound effect on which serovar was the second most commonly identified. In each model, this changed from Icterohaemorrhagiae to Grippotyphosa when comparing most positive titers to the highest titers, and finally when looking at titers ≥800, Pomona was the second most common. Looking at the most represented serovar in different ways could potentially skew the way we interpret geographical differences. A potential downside to the current equine vaccine is that it is made from serovar Pomona, which may not be the most pathogenic equine serovar seen in every area, and as previously discussed, very little cross protection exists. Further, some horses had titers to multiple serovars. This may represent multiple infection of different strains or different cross-reactions between serovars from the same serogroups, which is a reported problem with the MAT [27, 29].

One limitation of this study was that we only collected one, free catch urine sample from each horse. Some reports have said that shedding of leptospires in urine may be intermittent, therefore some potential carriers may have been missed [14]. Multiple samples from each horse over time may be something to consider in the future, however this may be difficult in field conditions. Culture or advanced molecular diagnostics were also considered to definitively diagnose serovar, however were not performed in this study due to study size and availability. Further, as Hamond and others [14] suggested in a similar study using urine PCR, we did not check for potential PCR inhibitors in clinical samples of urine and some has been stored for up to 48 hours at 4°C; Therefore, their presence cannot be ruled out as the cause of MAT positive (high titers)/PCR-negative results, particularly in those horses who had reciprocal MAT titers of greater than or equal to 800 which could indicate active infection. We also did not check for potential contaminating bacteria and fungi, which may lead to a false positive PCR result. Another point to consider with PCR is methodology. There are a variety of different targets, but ideally they should be able to differentiate pathogenic from non-pathogenic strains of *Leptospira* spp. Recently, differences between the types of PCR assays and their respective targets has come into question, particularly when considering other spirochetes or bacterial and fungal contaminants causing false positive results. In a study by Fink and others from 2015 [30], the performance of 3 real time PCR (qPCR) assays was assessed, 1 targeting the 16S ribosomal RNA (rRNA) gene and the other 2 targeting the lipL32 gene. When using DNA extracted from laboratory-cultured pathogenic *Leptospira* spp., all 3 assays demonstrated 100% specificity and had identical lower limits of detection when tested on urine samples collected aseptically from 30 dogs suspected for leptospirosis. However, when tested on 30 urine samples that were collected by the free-catch method, the 16S rRNA–based assay falsely detected 13.3% of the samples as positive for pathogenic *Leptospira* spp., yet nucleotide sequence analysis of the amplified DNA fragments showed that these were false positives due to unrelated bacteria [30]. These results highlight the importance of validated, sample-specific PCR-based diagnostic assays, that the use of 16S rRNA based assays may lead to increased false positive results, and that sampling method (free-catch vs aseptic) may have an effect on diagnosis. However, our PCR (23S rRNA based), unlike the 16S rRNA-based PCR, is less likely to detect non-pathogenic leptospiral DNA, similar to the LipL32 based assay [30]. Furthermore, an unpublished comparative study involving the Animal Disease Diagnostic Laboratory (Indiana, USA) and the Center for Disease Control (Georgia, USA) assays targeting the lipL32 gene versus the 23S rDNA target was performed at the Kansas State Molecular Diagnostic Laboratory. This study identified that the 23S rDNA target-based assay described in the present study was more sensitive (100%) compared to either of the real-time PCR assays targeting the lipL32 gene. (96.38% and 94.58%, respectively), out of the 166 known positives and 36 known negatives included (data is currently available in the KSVDL Leptospiral validation protocol).

In conclusion, the results of this study show that Central Midwestern horses are commonly exposed to pathogenic *Leptospira* spp. with exposure being most common to serovars belonging to Australis. Depending of evaluation method used, Grippotyphosa, Icterohaemorrhagiae, and Pomona were also serovars of interest. Rain in the horse’s environment during the previous week, as well as seasonality (summer and fall) seem to be potential risk factors for seropositivity (reciprocal MAT titer ≥ 100). Based on our findings, the risk of apparently healthy horses contributing to the spread of pathogenic *Leptospira* spp. in the environment appears low (1%). Future studies should focus on testing with serovar specificity, a wider geographical area and more risk factors, and horses with signs of clinical leptospirosis.
